# Association of Hypothyroidism With Low Serum Ferritin Levels and Iron-Deficiency Anemia During the First Trimester of Pregnancy

**DOI:** 10.7759/cureus.28307

**Published:** 2022-08-23

**Authors:** Vinayagamoorthi R, Pooja Dhiman, Rupavani Kollipaka, Sabita P, Hemavathy V

**Affiliations:** 1 Biochemistry, Indira Gandhi Medical College and Research Institute, Puducherry, IND; 2 Obstetrics and Gynaecology, Indira Gandhi Medical College and Research Institute, Puducherry, IND

**Keywords:** hemoglobin, iron-deficiency anemia, ferritin, hypothyroidism, pregnancy

## Abstract

Background

The association between hypothyroidism and iron-deficiency anemia (IDA) in pregnancy is not well established. Hence, this study aimed to investigate the association between hypothyroidism and IDA during the first trimester of pregnancy.

Methodology

In this study, a total of 144 pregnant women were included. Thyroid-stimulating hormone (TSH), free T4 (FT4), free T3 (FT3), and ferritin were measured. Based on TSH values, pregnant women were divided into the following two groups: euthyroid (n = 74) and hypothyroid (n = 70).

Results

There was a significant increase in TSH levels and a significant decrease in the levels of FT4, FT3, ferritin, iron, and hemoglobin (Hb) in hypothyroid pregnant women compared to euthyroid pregnant women. The correlation and regression analysis revealed a significant negative association of TSH and a positive association of FT4 with ferritin, iron, and Hb.

Conclusions

These findings demonstrate the association of hypothyroidism with IDA during the first trimester of pregnancy. Further studies with thyroxine therapy in hypothyroid pregnant women and its impact on IDA will open novel therapeutic approaches in the management of IDA during pregnancy. Further, measurement of serum ferritin during pregnancy may provide valuable information in the diagnosis and management of IDA.

## Introduction

Hypothyroidism is the most prevalent endocrine disorder in pregnant women. Changes in thyroid function during pregnancy are due to the influence of human chorionic gonadotropin (HCG) and estrogen [1]. High concentrations of HCG in the first trimester lower thyroid-stimulating hormone (TSH) levels and decrease the secretion of hormones by the thyroid gland [2]. Estrogen increases the concentration of circulating thyroid-binding proteins and lowers the functional free thyroid hormone concentrations [3]. Hypothyroidism causes many risks to pregnant women, including miscarriage, anemia, myopathy, pre-eclampsia, placental abnormalities, and postpartum hemorrhage [4]. During the first 18-20 weeks of pregnancy, the fetus is completely dependent on maternal thyroid hormones for its growth and development. Hypothyroidism in the mother can lead to impaired brain development in the baby [5]. Hence, early diagnosis and treatment for hypothyroidism are essential during pregnancy to prevent adverse effects on both the mother and the fetus.

Iron-deficiency anemia (IDA) is a widespread nutritional disorder and accounts for 75% of all types of anemia during pregnancy [6]. Pregnant women are highly susceptible to IDA due to their increased iron requirement. In pregnant women with iron deficiency, iron stored in bone marrow is mobilized to meet the demands. This depleted iron storage is reflected in serum ferritin levels [7]. IDA in pregnancy leads to several adverse consequences for both the mother and the fetus. Pregnant women with IDA have an increased risk for placental insufficiency, cardiac failure, and postpartum hemorrhage [8]. In babies, maternal IDA causes low birth weight and preterm complications [9]. Hence, early detection and treatment are necessary to prevent maternal and fetal morbidity and mortality associated with IDA.

Numerous studies have shown the association between thyroid disorders and IDA in animals and humans [10-12]. Studies have documented that hypothyroidism is associated with IDA in humans [13,14]. Though several studies have been conducted on hypothyroidism and IDA in pregnancy, the association between hypothyroidism and IDA in pregnancy is not well established. The limited studies also yielded contradictory reports. Pregnancy being an unique state, the outcomes of studies conducted in non-pregnant subjects cannot be extrapolated. Hence, it is necessary to establish the association between hypothyroidism and IDA in pregnancy. Further, such studies will help us understand their impact on pregancy and devise strategies for early diagnosis and better management to prevent maternal and fetal morbidity and mortality. In view of the above, this study was undertaken to investigate the association between hypothyroidism and IDA during the first trimester of pregnancy.

## Materials and methods

Study population

This study was conducted at the Indira Gandhi Medical College and Research Institute, Puducherry, India. Pregnant women attending the obstetric outpatient department for the first time during their first trimester (gestational age ranging between the first and the end of the twelfth week) were enrolled in this study.

Inclusion and exclusion criteria

Patients aged between 21 and 35 years, with a singleton pregnancy, normal thyroid peroxidase antibody (TpoAb < 9.0 IU/mL) levels, and normal anti-thyroglobulin antibody titer (TgAb < 116 IU/mL) were included in this study. We excluded patients with multiple pregnancies, anemia before pregnancy, those with known thyroid diseases, and those with other chronic illnesses, including diabetes, hypertension, malignancy, tuberculosis, human immunodeficiency virus, hepatitis B surface antigen, and treated with drugs that may affect thyroid function (glucocorticoids, dopamine, etc.). All procedures performed in this study were approved by the Human Ethical Committee of Indira Gandhi Medical College and Research Institute, Puducherry, India (283/IEC-30/IGMC&RI/PP/2020). Written informed consent was obtained from all participants included in this study.

Sample collection and analysis

A total of 144 pregnant women were included in this study. In total, 5 mL of blood samples were collected after overnight (minimum of eight hours) fasting. Serum TSH, free T4 (FT4), free T3 (FT3), TPOAb, TgAb, and ferritin were measured by chemiluminescence immunoassay using a CL900i analyzer (Mindray, Shenzhen, China). Serum iron was estimated using a fully automated analyzer (240pro; Mindray, Shenzhen, China), and hemoglobin (Hb) levels were estimated in ethylenediaminetetraacetic acid blood using an automated hematology analyzer (XP-300; Sysmex, Kobe, Japan).

Statistical analysis

Based on serum TSH levels, pregnant women were divided into the following two groups: euthyroid (TSH < 5.0 mIU/L, n = 74) and hypothyroid (TSH > 5.0 mIU/L, n = 70). Data were tabulated as mean ± standard deviation (SD). Differences between the two groups were analyzed by Student’s t-test. Pearson correlation analysis and linear regression analysis were used to establish the relationship between parameters. P-values of <0.05 were considered statistically significant. SPSS 26.0 (IBM Corp., Armonk, NY, USA) was used for the statistical analysis.

## Results

In this study, pregnant women were divided into euthyroid and hypothyroid groups, and their clinical parameters were assessed (Table 1). TSH levels were significantly (p < 0.001) increased in hypothyroid women compared to euthyroid women during their first trimester of pregnancy. Serum FT4 and FT3 levels were significantly (p < 0.001) decreased in hypothyroid pregnant women compared to euthyroid pregnant women. Hypothyroid pregnant women had a significantly (p < 0.05) higher body mass index (BMI) compared to eythyroid pregnant women. Ferritin (p < 0.001), Fe (p < 0.001), and Hb (p < 0.05) were significantly decreased in hypothyroid pregnant women compared to euthyroid pregnant women.

**Table 1:** Comparison of clinical parameters between euthyroid and hypothyroid pregnant women. *P-value < 0.05; **P-value <0.001.

Parameters	Euthyroid	Hypothyroid
Numbers	74	70
Age (years)	25.45 ± 2.78	25.84 ± 2.88
Gestational age (weeks)	10.18 ± 1.13	10.48 ± 1.15
Basal metabolic rate (kg/m^2^)	21.52 ± 1.80	25.02 ± 1.79*
Thyroid peroxidase antibody (IU/mL)	1.37 ± 0.80	1.40 ± 0.84
Anti-thyroglobulin antibody (IU/ml)	56.78 ± 12.73	48.78 ± 14.44
Thyroid-stimulating hormone (mIU/L)	3.11 ± 0.83	35.70 ± 13.84**
Free T4 (ng/dL)	0.91 ± 0.25	0.41 ± 0.30**
Free T3 (pg/mL)	2.96 ± 0.80	0.69 ± 0.34**
Ferritin (µg/L)	174.93 ± 37.70	102.91 ± 27.47**
Iron (µg/L)	123.56 ± 13.96	57.94 ± 21.07**
Hemoglobin (g/dL)	11.98 ± 1.28	7.06 ± 0.89*

The distribution of Hb concentration among hypothyroid pregnant women is presented in Table 2. Out of 70 hypothyroid pregnant women included in this study, 34 (49%) had Hb concentrations less than 8 g/dL, 30 (42%) had Hb between 8 g/dL and 10 g/dL, and six (9%) had Hb concentrations more than 10 g/dL. Interestingly, this data showed that out of 70 hypothyroid pregnant women included in the present study, 64 (91%) showed Hb concentrations below 10 g/dL.

**Table 2:** Hemoglobin concentration among hypothyroid pregnant women.

Distribution of hemoglobin concentration	Number of hypothyroid pregnant women (N = 70)	Percentage (%)
Hemoglobin <8 (g/dL)	34 (70)	49%
Hemoglobin 8–10 (g/dL)	30 (70)	42%
Hemoglobin >10 (g/dL)	06 (70)	09%

**Figure 1:**
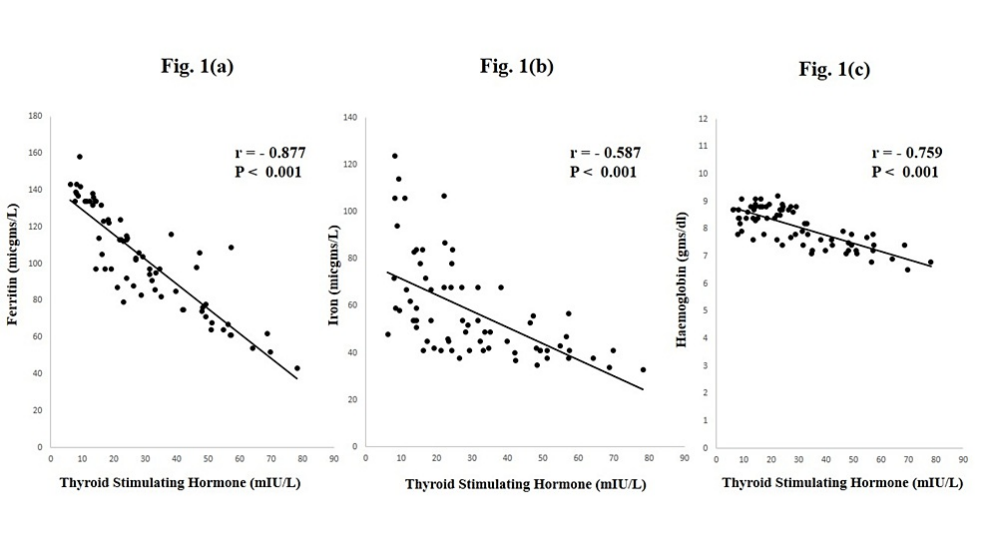
Relationship between thyroid-stimulating hormone and hematological parameters in hypothyroid pregnant women.

Pearson correlation analysis showed that TSH levels were negatively correlated (Figure 1) with with ferritin (r = -0.877, p < 0.001) (Figure 1a), Fe (r = -0.587, p < 0.001) (Figure 1b), and Hb (r = -0.759, p < 0.001) (Figure 1c).

**Figure 2:**
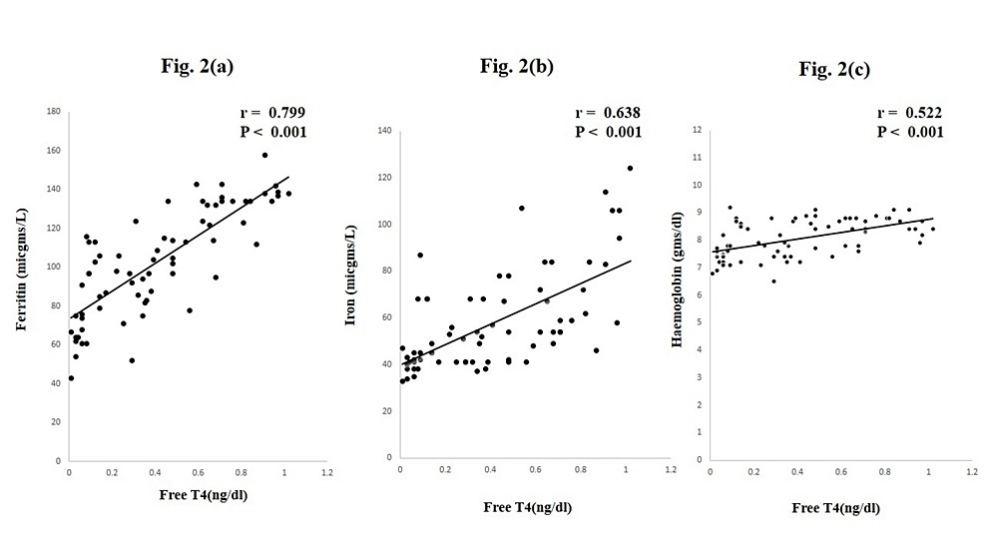
Relationship between free T4 and hematological parameters in hypothyroid pregnant women.

Pearson correlation analysis revealed a positive correlation (Figure 2) of FT4 levels with ferritin (r = 0.799, p < 0.001) (Figure 2a), Fe (r = 0.638, p = 0.006) (Figure 2b), and Hb (r = 0.522, p < 0.001) (Figure 2c).

TSH as predictor showed (Table 3) a negative correlation with ferritin (β = -0.638, p < 0.001), Fe (β = -0.390, p < 0.001), and Hb (β = -0.854, p < 0.001) included as dependent variables. FT4 as predictor showed a positive correlation with ferritin (β = 0.355, p < 0.001), Fe (β = 0.458, p < 0.001), and Hb (β = 0.204, p = 0.003) included as dependent variables. These results clearly suggest that TSH had a negative and FT4 had a positive correlation with hematological parameters such as ferritin, Fe and Hb. These observations indicate that, in pregnant women during their first trimester, hypothyroidism is associated with IDA.

**Table 3:** Linear regression between thyroid-stimulating hormone or free T4 with ferritin, Fe, and hemoglobin in hypothyroid pregnant women.

	Ferritin (dependent variable)		Iron (dependent variable)		Hemoglobin (dependent variable)	
	β value	P-value	β value	P-value	β value	P-value
Thyroid-stimulating hormone (predictor)	-0.638	<0.001	-0.390	0.006	-0.854	<0.001
Free T4 (predictor)	0.355	<0.001	0.458	<0.001	0.204	0.003

## Discussion

This study aimed to investigate the association between hypothyroidism and IDA during the first trimester of pregnancy. A total of 144 pregnant women were included, and TSH, FT4, FT3, TPOAb, TgAb, ferritin, Fe, and Hb were measured. Based on their TSH values, pregnant women were divided into two groups, namely, euthyroid (n = 74) and hypothyroid (n = 70). There was a significant increase in TSH levels and a significant decrease in the levels of FT4, FT3, ferritin, Fe, and Hb in hypothyroid pregnant women compared with euthyroid pregnant women. The correlation and regression analysis revealed a significant negative association of TSH and a positive association of FT4 with ferritin, Fe, and Hb. These findings clearly show the association of hypothyroidism with IDA in pregnancy.

Previous studies have documented the association of thyroid function with anemia [15,16]. Studies have shown hypothyroidism as one of the most important causes of IDA [17,18]. It has been proved that thyroxine therapy improves IDA [19]. However, all these studies were conducted in non-pregnant subjects. The reports of non-pregnant subjects cannot be extrapolated to pregnant women because pregnancy is a unique nutritional, physiological, metabolic, and hormonal state. To our knowledge, for the first time, we have investigated the association of hypothyroidism with IDA during the first trimester of pregnancy. Our results indicate that hypothyroidism is strongly associated with IDA during the first trimester of pregnancy. Our findings in pregnant women match with the outcomes of studies conducted among non-pregnant subjects.

**Figure 3:**
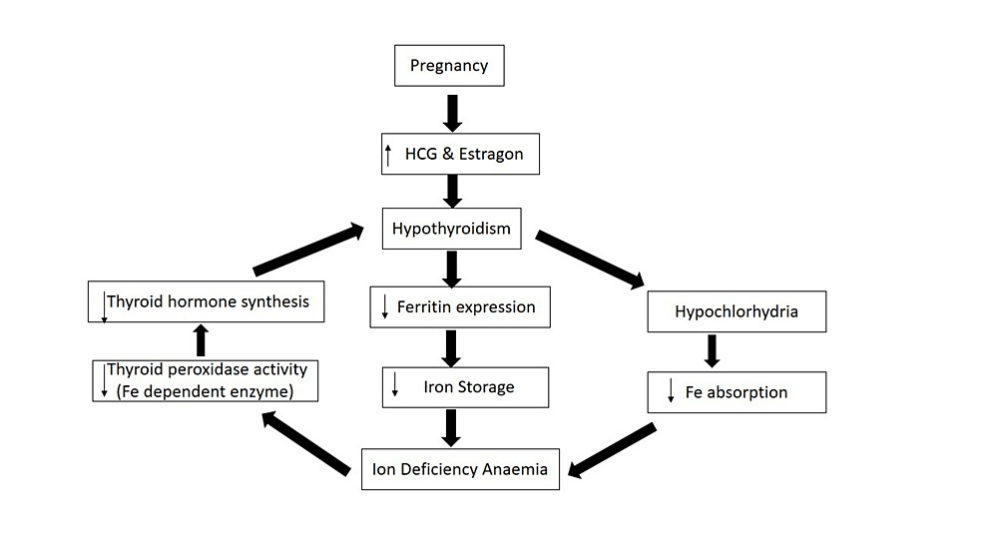
Proposed mechanisms for hypothyroidism-induced iron-deficiency anemia in pregnancy. HCG: human chorionic gonadotropin

Numerous mechanisms have been advocated for IDA in hypothyroidism. Studies have revealed the role of thyroid hormones in the regulation of ferritin expression [20,21]. Thyroid hormone binding elements are documented upstream of the ferritin gene, and the binding of thyroid hormones to these elements enhances ferritin synthesis [22]. One possible explanation for IDA in hypothyroidism could be due to less ferritin synthesis, which leads to low serum levels of ferritin, as seen in our hypothyroid pregnant women. Because ferritin is an iron storage protein, low levels of ferritin decrease iron storage in the body. Due to increased iron requirements in pregnancy, the limited stored iron also mobilized and led to IDA. Secondary to low ferritin levels, hypothyroidism causes hypochlorhydria, and decreases dietary iron absorption, which leads to iron deficiency. Thyroid peroxidase (TPO), an iron-containing enzyme, plays a key role in the synthesis of thyroid hormones [23]. Iron deficiency due to low ferritin and hypochlorhydria in hypothyroidism decreases TPO activity and further impairs thyroid function. Hence, we propose that decreased expression of ferritin, hypochlorhydria, and decreased TPO activity are the possible mechanisms for hypothyroidism-induced IDA in pregnancy (Figure 3).

## Conclusions

Our study for the first time showed the association of hypothyroidism with IDA during the first trimester of pregnancy. Further studies with larger sample sizes and thyroxine therapy in hypothyroid pregnant women and its impact on IDA will open new therapeutic strategies in the management of IDA during pregnancy. Besides, the measurement of serum ferritin during pregnancy may provide valuable information in the diagnosis and management of IDA.
